# Mapping the Plasticity of Morphology, Molecular Properties and Function in Mouse Primary Microglia

**DOI:** 10.3389/fncel.2021.811061

**Published:** 2022-01-26

**Authors:** Xue Jiang, Hui He, Li Mo, Qin Liu, Fan Yang, Ying Zhou, Liangyuan Li, Dapeng Su, Saini Yi, Jinqiang Zhang

**Affiliations:** ^1^Resource Institute for Chinese and Ethnic Materia Medica, Guizhou University of Traditional Chinese Medicine, Guiyang, China; ^2^State Key Laboratory of Quality Research in Chinese Medicine, Institute of Chinese Medical Sciences, University of Macau, Macau, China; ^3^School of Life Sciences and Technology, University of Electronic Science and Technology of China, Chengdu, China; ^4^Second Affiliated Hospital, Guizhou University of Traditional Chinese Medicine, Guiyang, China; ^5^Guizhou Provincial People’s Hospital, Guiyang, China

**Keywords:** microglia, phenotypes, morphology, phagocytosis, neurotoxicity, plasticity

## Abstract

Microglia exert diverse functions by responding in diverse ways to different stimuli, yet little is known about the plasticity of various phenotypes that microglia display. We used interferon (IFN)-γ, interleukin (IL)-4 and IL-10 to induce different phenotypes in mouse primary microglia. RNA sequencing was used to identify genes differentially expressed in response to stimulation, and the different stimulated populations were compared in terms of morphology, proliferative capacity, phagocytic ability and neurotoxicity. IFN-γ induced an “immunodefensive” phenotype characterizing both induction of filopodia and upregulation of inducible nitric oxide synthase (iNOS) and tumor necrosis factor α. Microglia with this phenotype mediated an acute inflammatory response accompanied by excellent proliferative capacity and neurotoxicity, and remained susceptible to remodeling for up to 48 h after initial stimulation. IL-4 induced an enduring “neuroimmunoregulatory” phenotype involving induction of lamellipodium and persistent upregulation of arginase (Arg)-1 and YM-1 expression. Microglia with this phenotype remained susceptible to remodeling for up to 24 h after initial stimulation. IL-10 induced an “immunosuppressive” phenotype involving induction of ameba-like morphology and upregulation of transforming growth factor β and IL-10 as well as inhibition of inflammation. This phenotype was accompanied by inhibition of self-proliferation, while its morphology, molecular properties and function were the least susceptible to remodeling. IFN-γ, IL-4, or IL-10 appear to induce substantially different phenotypes in microglia. The immunodefensive microglia induced by IFN-γ showed remarkable plasticity, which may help repair CNS inflammation damage under pathological condition. Chronic activation with IL-10 decreases microglial plasticity, which may help protect the brain form the immune response. Our research justifies and guides further studies into the molecular pathways that operate in each phenotype to help multitasking microglia regulate homeostasis in the brain.

## Introduction

Microglia play crucial roles in the development and homeostasis of the central nervous system, as well as in its acute responses to infection or injury (Tambuyzer et al., 2009). To perform their diverse functions, microglia adopt diverse phenotypes (Stratoulias et al., 2019). Understanding how microglia integrate signals to adopt different phenotypes is important not only for appreciating their polymorphic, multilayered nature, but also for identifying therapeutic strategies to limit excessive inflammation in neuroinflammatory diseases.

The phenotypic plasticity of microglia can be observed at the morphological level (Sankowski et al., 2019). Interleukin 4 stimulation, immediately following lipopolysaccharide stimulation, re-multibranched ameba-like mouse microglia (Mecha et al., 2015; Nguyen et al., 2017). Our previous studies have also found that ameboid microglia isolated from postnatal (P0–P3) mice showed increased ramification when cultured in astrocyte-conditioned medium or on astrocyte monolayers (Zhang et al., 2020c). The number, length of branches and body size of microglia can be manipulated *in vivo* and *in vitro* by regulating interferon regulatory factors (IRFs), such as IFN regulatory factor 8 (IRF8) (Horiuchi et al., 2012; Masuda et al., 2012; Minten et al., 2012).

The phenotypic plasticity of microglia can be observed at the level of gene expression and protein secretion (Chhor et al., 2013; Vay et al., 2018; Bisicchia et al., 2019). Anti-inflammatory cytokines could reprogram the microglia responses to pro-inflammatory stimuli (Fumagalli et al., 2018; Waltl and Kalinke, 2021; Yang et al., 2021). Interleukin-10, interleukin-4, and transforming growth factor-beta differentially regulate lipopolysaccharide-induced production of pro-inflammatory cytokines and nitric oxide in microglial cells (Ledeboer et al., 2000). Emerging strategies able to redirect microglia from detrimental to beneficial phenotype is attractive, opening novel approaches to target microglia therapeutically (Zhang et al., 2021). Less clear about phenotypic plasticity is what particular morphological and gene expression signatures may be associated with particular downstream effects, such as pro- or anti-inflammatory responses. Also unclear is how easily microglia with a given phenotype can switch to a different phenotype, which is crucial for responding quickly to changing environments.

Here we examined the plasticity of primary mouse microglia in terms of morphology, gene expression and function. Although the *in vitro* model has some limitations, this work may help clarify the diversity of microglia in complex brain environments and preliminarily identify molecular pathways that contribute to neurological disease.

## Materials and Methods

### Primary Microglial Culture

Cerebral hemispheres were obtained from male C57BL/6J mice within the same litter on postnatal days 1–3. Tissue was taken from one sex to reduce confounding. Cerebellum and olfactory bulb was excised from cerebral hemispheres in sterile phosphate-buffered saline (PBS) to allow stripping of the meninges. Tissue was minced on ice, digested using 0.25% pancreatin, and filtered through a 70-μm cell strainer to yield a single-cell suspension. The suspension was cultured in DMEM/F12 (Gibco, Grand Island, NY, United States) containing 10% fetal bovine serum (Gibco) at 37°C in an atmosphere of 5% CO_2_. This procedure gave rise to mixed cultures in which microglia grew loosely atop a layer of tightly adhering astrocytes. Microglia were harvested by mechanical shaking, then transferred to new culture dishes. The purity of microglia is verified using immunofluorescence. Microglia were labeled with Iba1 antibodies, and the nucleus were labeled with DAPI. More than 98% of the Iba1^+^ cells were used in flowing plasticity research.

### Stimulation of Primary Microglia

Interleukin (IL)-4, IL-10 and interferon (IFN)-γ (PeproTech, Rocky Hill, NJ, United States) were diluted in PBS and added to primary cultures of microglia at final respective concentrations of 20, 20, and 50 μg/mL. Cells were treated for 2, 4, 6, 12, 24, 48, and 72 h. At these time points, microglial phenotype was assessed in terms of morphology proliferation ability and gene expression as described below.

In experiments to assess how easily microglia with one phenotype could be switched to another phenotype, cells were exposed to IFN-γ, IL-4, or IL-10 for 2–72 h, then treated with one cytokine for 2–72 h, then the medium was changed and the cells were stimulated with another cytokine for 12 h. Control cells were re-stimulated with the same cytokine as initially. And cells assessed in terms of morphology, proliferation ability and gene expression as described below.

### RNA Sequencing

Primary microglia were incubated for 24 h with each of the three cytokines at the concentrations indicated in Section “Stimulation of Primary Microglia,” then total RNA was extracted using TRIzol^®^ Reagent according to the manufacturer’s instructions (Invitrogen, Carlsbad, CA, United States). Genomic DNA was removed using DNase I (TaKara, Tokyo, Japan), and RNA quality was assessed using a 2100 Bioanalyser (Agilent, Santa Clara, CA, United States) and a ND-2000 system (NanoDrop Technologies, WTN, United States). Only RNA samples giving optical density ratios of 1.8–2.2 (260/280 nm) and optical purity ratios ≥ 2.0 (260/230 nm) as well as an RNA integrity number ≥ 6.5 and a 28S:18S ratio ≥ 1.0 were used to construct the sequencing library.

An RNA-seq transcriptome library was prepared from 1 μg of total RNA using the TruSeq™ RNA sample preparation kit (Illumina, San Diego, CA, United States). Briefly, mRNA was isolated using oligo (dT) beads, fragmented, and used to prepare double-stranded cDNA using a Super Script kit (Invitrogen, Carlsbad, CA, United States) and random hexamer primers (Illumina, CA, United States). Then the synthesized cDNA was subjected to end-repair, phosphorylation and ‘A’ addition according to the Illumina library construction protocol. The cDNA fragments of 200–300 bp were selected by electrophoresis on 2% Low Range Ultra Agarose (Bio-Rad, Hercules, CA, United States), then amplified in 15 cycles of PCR with Phusion DNA polymerase (NEB, Shanghai, China). Amplicons were quantified using TBS380 (Turner Biosystems, Sunnyvale, CA, United States), and the paired-end RNA-seq library was sequenced on an Illumina NovaSeq 6000 system (2 × 150 bp read length). We have submitted our RNA-Seq data to the NCBI database (Submission ID: SUB10788230; BioProject ID: PRJNA787892).

### Analysis of RNA Sequencing Data

RNA sequencing reads were removed if they contained adaptors, more than 10% unknown bases or more than 50% bases of low-quality. Based on the resulting high-quality data, the level of each transcript was calculated according to the “fragments per kilobase of exon per million mapped reads” method.

Differentially expressed genes (DEGs) among the microglia stimulated with different cytokines were identified using the “Empirical Analysis of Digital Gene Expression in R” (EdgeR) software. Enrichment of DEGs in certain Gene Ontology (GO) categories or certain Kyoto Encyclopedia of Genes and Genomes (KEGG) pathways was analyzed using, respectively, Goatools and Kobas.

### Immunofluorescence

Primary microglia were seeded in quadruplicate on glass coverslips (1 × 10^5^ cells per mm^2^). Cells were washed with PBS, incubated with 4% paraformaldehyde (PFA) for 30 min, blocked for 2 h with 10% bovine serum albumin, then labeled overnight using goat primary antibodies against Iba1 or rabbit primary antibodies against Arginase (Arg)-1, inducible nitric oxide synthase (iNOS), transforming growth factor (TFG)-β1 or tubulin. All antibodies were purchased from Abcam (Cambridge, MA, United States) and used at 1:400 dilution. Cells were then stained with one of the following donkey secondary antibodies (1:500; Jackson ImmunoResearch, West Grove, PA, United States): DyLight 488-conjugated anti-mouse antibody, DyLight 549-conjugated anti-goat antibody, or DyLight 488-conjugated anti-rabbit antibody. Finally, cells were stained for 5 min with DAPI (1:10,000; Roche, Switzerland) and imaged using a fluorescence microscope (Olympus BX51, Japan).

### Analysis of Microglial Morphology

Images (40×) were analyzed by another experimenter who were double-blinded using Image J software (version 1.45J; National Institutes of Health, Bethesda, MD, United States). A staining intensity threshold was defined, and the percentage of the coverslip area showing at least this intensity was determined. Microglial images were adjusted to observe all microglial processes via standard background subtraction (50 pixels with the sliding parabola option), and single-pixel background fluorescence was eliminated. Then, resulting images were converted to a binary image and skeletonized. The “Analyze Skeleton” plugin was used to analyze all skeletonized images, and soma area, number of somata per field and diameter of soma were determined. At least nine random fields on at least four separate coverslips were analyzed for each treatment.

### RT-PCR

RNA was isolated from primary microglia using Trizol reagent (Life Technologies, CA, United States) and chloroform extraction, purified with the Qiagen RNeasy kit (Takara, Japan), and reverse-transcribed into cDNA using a high-capacity cDNA conversion kit (Takara, Japan). Quantitative RT-PCR was performed on a Bio-Rad CFX 96 system (Hercules, CA, United States) using the following primers: β-actin forward, 5′-CCGTGAAAAGATGACCCAGATC-3′; β-actin reverse, 5′-CA CAGCCTGGATGGCTACGT-3′; TNF-α forward, 5′-TACTGAA CTTCGGGGTGATTGGTCC-3′; TNF-α reverse, 5′-CAGCCTT GTCCCTTGAAGAGAACC-3′; iNOS forward, 5′-ACAACAGG AACCTACCAGCTCA-3′; iNOS reverse, 5′-GATGTTGTAGC GCTGTGTGTCA-3′; IL-10 forward, 5′-TGGCCCAGAAA TCAAGGAGC-3′; IL-10 reverse, 5′-CAGCAGACTCAATAC ACACT-3′; TGF-β forward, 5′-GACCGCAACAACGCCATCTA-3′; TGF-β reverse, 5′-GGCGTATCAGTGGGGGTCAG-3′; Arg-1 forward, 5′-AGACAGCAGAGGAGGTGAAGAG-3′; Arg-1 reverse, 5′-CGAAGCAAGCCAAGGTTAAAGC-3′; YM-1 forward, 5′-GGGCATACCTTTATCCTGAG-3′; and YM-1 reverse, 5′-CCACTGAAGTCATCCATGTC-3′.

Threshold amplification cycle numbers (Ct) were determined from the linear phase of the amplification plot. Each sample was tested in triplicate. Levels of transcripts were determined using the −ΔΔCt method and normalized to levels of β-actin.

In experiments to track changes in microglial phenotype markers over time, primary microglia were treated with each of the three cytokines for 0–72 h. At various time points, total RNA was isolated and marker gene expression was analyzed by RT-PCR as described above.

### Enzyme-Linked Immunosorbent Assay

Levels of iNOS, Arg-1 and TGF-β in primary microglia as well as levels of IL-1β, IL-6, and TNF-α in culture medium were quantified using commercial ELISA kits for mouse tissue (QuantiCyto, Wuhan, China) according to the manufacturer’s protocols. Absorbance was measured at 450 nm using a microplate reader, then converted to pg/mL.

### Nitric Oxide Assay

Nitric oxide (NO) secreted by primary microglial cultures was assayed indirectly using a commercial kit that measured levels of its stable metabolites, nitrite and nitrate (Total Nitric Oxide and Nitrate/Nitrite Assay, R&D Systems, Minneapolis, MN, United States). The kit was used according to the manufacturer’s recommendations.

### Bromodeoxyuridine Labeling

Microglial cultures were treated with each of the cytokines as described in Section “Stimulation of Primary Microglia,” after which 100 ng/mL BrdU was added to the medium. At 2–72 h later, microglia were fixed for 30 min in 4% PFA, blocked with 10% bovine serum albumin, then labeled overnight using the same goat anti-Iba1 antibody as in Section “Immunofluorescence” and mouse anti-BrdU antibody (1:400; Cell Signaling Technology, United States), followed by the anti-mouse and anti-goat secondary antibodies mentioned in Section “Immunofluorescence”. Finally, cells were stained for 5 min with DAPI (1:10,000) and imaged using a fluorescence microscope (Olympus BX51, Japan). Cells positive for BrdU and Iba1 were considered to be proliferating microglia.

### Assay of Phagocytic Ability

Microglia were seeded into 24-well plates at a density of 2 × 10^5^ cells/cm^2^, cultured for 90 min at 37°C, and then microbeads with a diameter of 0.6 μm (Bio-Rad, United States) were added to each well (2 × 10^5^ beads per well). Cells were washed with PBS for 15 min, fixed with PFA for 1 h, then stained with the same goat anti-Iba1 antibody as in Section “Immunofluorescence.”

### Culture of Neural Stem/Progenitor Cells With Conditioned Medium From Cytokines-Treated Microglia

Neural stem/progenitor cells (NSPCs) were generated from the subependymal ventricular zone (SVZ) of young adult mice and cultured under clonal conditions, according to previously published procedures (Zhang et al., 2021). Microglia were plated at a density of 5 × 10^5^ cells/cm^2^ and treated with each of the cytokines as described in Section “Stimulation of Primary Microglia,” followed by washing with PBS twice, and then addition of DMEM-F12 + GlutaMax medium for another 24 h. The microglial medium was collected and used as conditioned medium to culture NSPCs for 24 h. Apoptosis of NSPCs were labeled overnight using the mouse anti-cleaved caspase-3 antibody (1:300; Cell Signaling Technology, United States), followed by the anti-mouse secondary antibodies mentioned in Section “Immunofluorescence.” Finally, cells were stained for 5 min with DAPI (1:10,000) and imaged using a fluorescence microscope (Olympus BX51, Japan).

### Statistical Analysis

Data were analyzed for normal distribution using the Shapiro–Wilk test, and all statistical tests were performed in GraphPad Prism (version 6.0, United States). Pairwise comparisons were assessed for significance using two-tailed *t*-test, and comparisons of three or more values were assessed using one- or two-way ANOVA and Tukey’s *post hoc* tests. Levels of significance are marked in figures as *p < 0.05, significant; ^**^p < 0.01, very significant; and ^***^p < 0.001, highly significant.

## Results

### Transcriptional Profiling of Primary Microglia Stimulated by IFN-γ, IL-4, or IL-10

**FIGURE 1:**
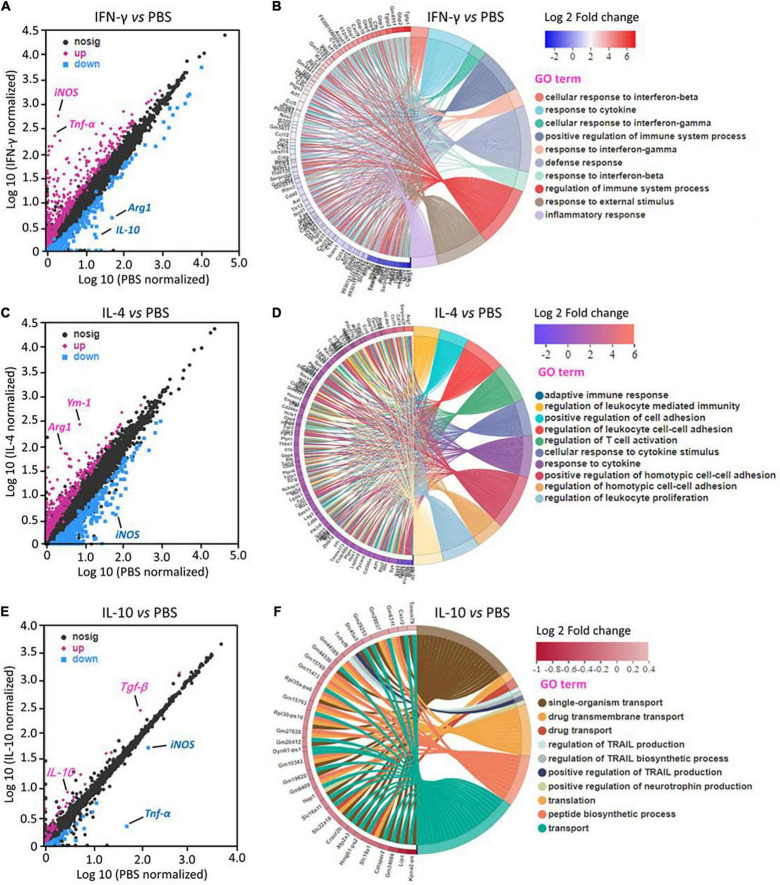
Transcriptional profiling of primary microglia treated with IFN-γ, IL-4, or IL-10. **(A,C,E)** Differentially expressed genes (DEGs) in primary microglia following treatment with IFN-γ, IL-4, or IL-10. Red DEGs are upregulated; blue DEGs, downregulated. **(B,D,F)** Enrichment of DEGs in Gene Ontology categories (*n* = 3).

RNA sequencing identified 210 DEGs in primary microglia that were upregulated by IFN-γ and 112 that were downregulated. The most strongly upregulated DEGs encoded iNOS and TNF-α. Most DEGs are involved in immune defense and inflammatory response (Figures 1A,B).

IL-4 upregulated 221 DEGs and downregulated 211. The most strongly upregulated encoded Arg-1 and Ym-1. Most DEGs are involved in immunomodulation (Figures 1C,D).

IL-10 upregulated 32 DEGs and downregulated 57. The most strongly upregulated encoded TGF-β and IL-10. Most DEGs are involved in immune deactivation and production of TNF-related apoptosis-inducing ligand (Figures 1E,F).

**FIGURE 2:**
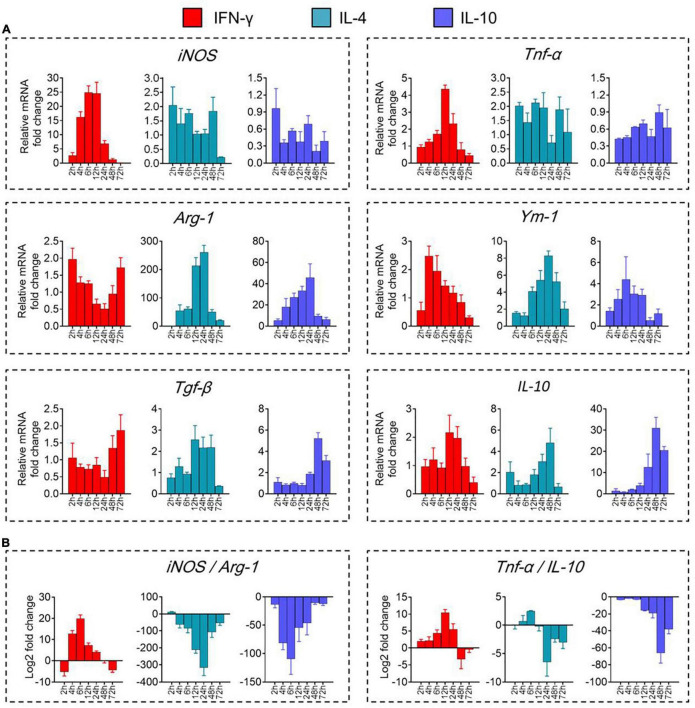
Transcriptional definitions of microglial phenotypes induced by IFN-γ, IL-4, or IL-10. **(A)** Levels of mRNAs encoding iNOS, TNF-α, Arg-1, Ym-1, Tgf-β, or IL-10 following treatment with each cytokine for 2–72 h. **(B)** Ratios of potential marker mRNAs differentiating the three microglial phenotypes: iNOS/Arg-1 and TNF-α/IL-10. Data are presented as mean ± SEM (*n* = 4–6 well per group, three replicates per sample).

We validated these results by showing that levels of mRNAs encoding the immune-defense-related markers iNOS and TNF-α were elevated during prometaphase at 2–24 h of IFN-γ treatment, peaking at 12 h (Figure 2A). Similarly, levels of mRNAs encoding the immunomodulation-related markers Arg-1 and Ym-1 were elevated between 4 and 48 h of IL-4 treatment, peaking at 24 h. Levels of mRNAs encoding the immune deactivation-related markers TGF-β and IL-10 were elevated between 24 and 72 h of IL-10 treatment, peaking at 48 h.

**FIGURE 3:**
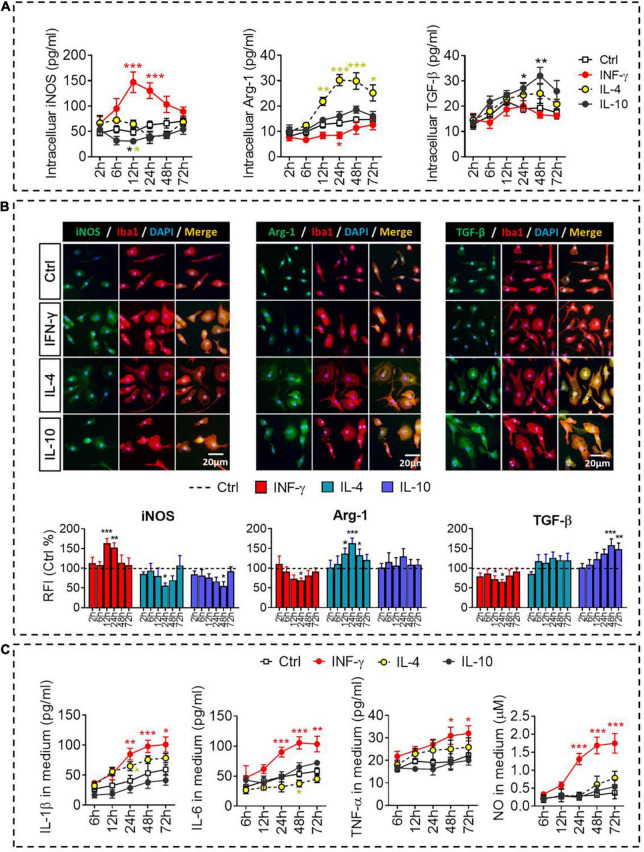
Identification of intracellular and extracellular proteins in IFN-γ-, IL- 4-, or IL-10-stimulated microglia. **(A)** Quantification of the intracellular protein concentration of iNOS, Arg-1, or TGF-β following treatment with IFN-γ, IL-4, or IL-10 for 2–72 h. **(B)** Fluorescence micrographs of primary microglial cultures treated for 24 h with each of the cytokines and then immunostained for Iba1 as well as iNOS, Arg-1, or TGF-β. The histogram represent quantification of the relative fluorescence intensity (RFI) of iNOS, Arg-1 or TGF-β following treatment with IFN-γ, IL-4, or IL-10 for 2–72 h. The RFI were normalized with Ctrl group. **(C)** Quantification of the inflammatory mediators (IL-β, TNF-α, and IL-6) and toxic molecules (NO) in medium following treatment with IFN-γ, IL-4, or IL-10 for 2–72 h. Data are presented as mean ± SEM (*n* = 4–6 well per group, three replicates per sample), *P < 0.05, **P < 0.01, ***P < 0.005 (one-way ANOVA with Tukey’s multiple-comparisons test).

The dynamic alteration of the immune-defense/immunomodulation or immune-defense/immune deactivation markers in primary microglia was characterized continuously by examining the ratios of iNOS/Arg-1 and TNF-α/IL-10, respectively. Treatment with IFN-γ for 12 h significantly increased the ratios of mRNAs encoding iNOS/Arg-1 and TNF-α*/*IL-10 in microglia. Treatment with IL-4 for 24 h significantly increased the ratio Arg-1/iNOS. Treatment with IL-10 for 48 h significantly increased the ratio IL-10/TNF-α (Figure 2B). These analyses at the mRNA level were confirmed at the protein level using ELISA (Figure 3A) immunocytochemistry (Figure 3B). IFN-γ increased the concentration of inflammatory mediators (IL-β, TNF-α, and IL-6) and toxic molecules (NO) in microglial medium after 24 h (Figure 3C).

These results suggest that the different cytokines induce transcription of different genes, which presumably leads to different functions. We reasoned that the DEGs most strongly upregulated by each cytokine may be useful for defining distinct microglial phenotypes.

### Morphological Analysis of Primary Microglia Stimulated by IFN-γ, IL-4, or IL-10

**FIGURE 4:**
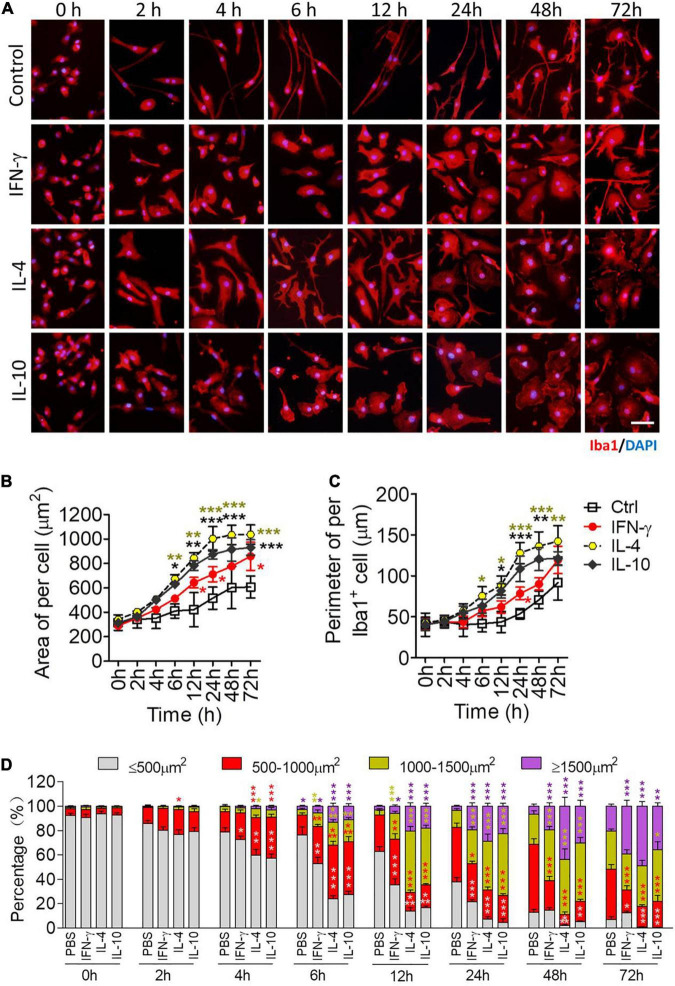
Morphological definition of microglial phenotypes induced by IFN-γ, IL-4, or IL-10. **(A)** Microglial morphology in response to treatment with IFN-γ, IL-4, or IL-10 for the indicated times. Microglia were immunolabeled for Iba1 (red), and nuclei were labeled with DAPI (blue). **(B)** Microglial area after treatment with IFN-γ, IL-4, or IL-10 treatment for 2–72 h. **(C)** Microglial diameter after treatment with IFN-γ, IL-4, or IL-10 for 2–72 h. **(D)** Size distribution of microglia after treatment with IFN-γ, IL-4, or IL-10 for 2–72 h. Five, 40× micrographs were taken from each well, and all cells in the field were measured. Data are presented as mean ± SEM (*n* = 4 well per group), *P < 0.05, **P < 0.01, ***P < 0.005 (one-way ANOVA with Tukey’s multiple-comparisons test).

Microglia were stimulated with each of the cytokines as described in Section “Stimulation of Primary Microglia,” then analyzed by microscopy at various time points between 2 and 72 h later. Before activation, most microglia were spindle-shaped with a small soma and two branches, one short and thick and the other long and thin, on opposite sides of the soma (Figure 4A). IFN-γ led to a rounded soma and multiple filopodia. IL-4 led to an irregular soma featuring 1–2 main lamellipodia as well as several filopodia. IL-10 caused retraction of processes, leading to a rounded, amoeboid cell.

Each of the cytokines led to an enlarged soma at 12–72 h, and IL-4 and IL-10 led to larger, thicker microglia than IFN-γ (Figures 4B,C). IL-4 or IL-10 increased the number of microglia with larger cell bodies after 6–72 h (Figure 4D). In general, IFN-γ induced smaller and slower morphological changes than the other cytokines (Figure 4D).

These morphological findings support the transcriptional profiling to suggest that each cytokine activates microglia to adopt a distinct phenotype.

### Proliferative Ability of Primary Microglia Stimulated by IFN-γ, IL-4, or IL-10

**FIGURE 5:**
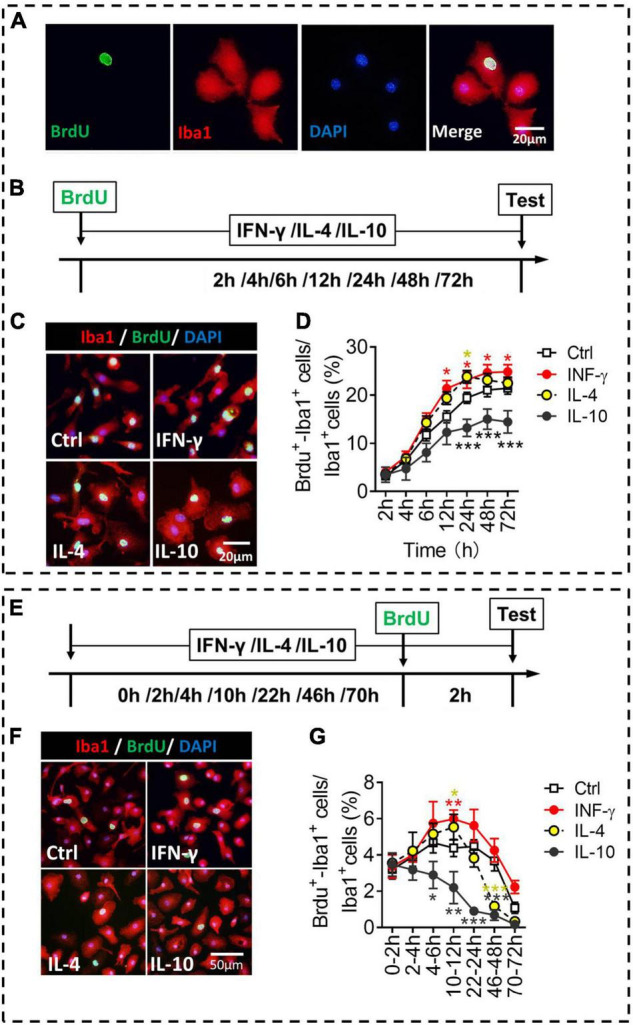
Proliferative ability of microglial phenotypes induced by IFN-γ, IL-4, or IL-10. **(A)** Representative micrographs showing immunocytochemical labeling for BrdU (green) and Iba1 (red) in microglia. Cells double-labeled with BrdU and Iba1 were considered to be proliferating microglia. **(B)** Experimental scheme to monitor proliferation ability during activation. **(C)** Representative micrographs showing immunocytochemical labeling for BrdU and Iba1 in microglia treated for 24 h with IFN-γ, IL-4, or IL-10. **(D)** Quantification of the percentages of BrdU^+^-Iba1^+^ cells following treatment with IFN-γ, IL-4, or IL-10 for 2–72 h. **(E)** Experimental scheme to monitor proliferation ability after activation for 2–72 h. **(F)** Representative micrographs showing immunocytochemical labeling for BrdU and Iba1 in microglia treated for 22 h with IFN-γ, IL-4, or IL-10, followed by treatment for 2 h with BrdU. **(G)** Quantification of the percentage of proliferating microglia during 2 h after treatment with IFN-γ, IL-4, or IL-10 for 2–72 h. Five, 40× micrographs were taken from each well, and all cells in the field were measured. Data are presented as mean ± SEM (*n* = 5–6 well per group), *P < 0.05, **P < 0.01, ***P < 0.005 (one-way ANOVA with Tukey’s multiple-comparisons test).

We compared the different phenotypes of microglia in terms of proliferation, an important indicator of activation (Figure 5A). Microglia were exposed to each of the three cytokines for 2–72 h, and numbers of BrdU^+^-Iba1^+^ cells were quantified (Figure 5B). IFN-γ increased, while IL-10 decreased, the numbers of proliferative microglia at 24–72 h of treatment. IL-4 increased the numbers of proliferative microglia at 24 h of treatment (Figures 5C,D).

To evaluate whether the microglial proliferation was synchronized with the changes in morphology and phenotypic marker expression, we compared their proliferation at different time periods after exposure of three cytokines (Figure 5E). The results showed that IFN-γ promoted self-proliferation of microglia at 12 h, after which it declined. Conversely, IL-10 inhibited the self-proliferation of microglia at 6–48 h. IL-4 promoted self-proliferation of microglia at 12 h, but inhibited the self-proliferation of microglia at 48 h after treatment (Figures 5F,G).

These findings suggest that IFN-γ, IL-4, and IL-10 differentially regulate proliferative ability of primary microglia. Not all microglial activation is accompanied by up-regulation of proliferation ability, for example, IL-10-stimulated microglia invariably shows downregulation in proliferative capacity. These findings are helpful to study the proliferative function of primary microglia *in vitro*.

### Phagocytic Ability of Primary Microglia Stimulated by IFN-γ, IL-4, or IL-10

**FIGURE 6:**
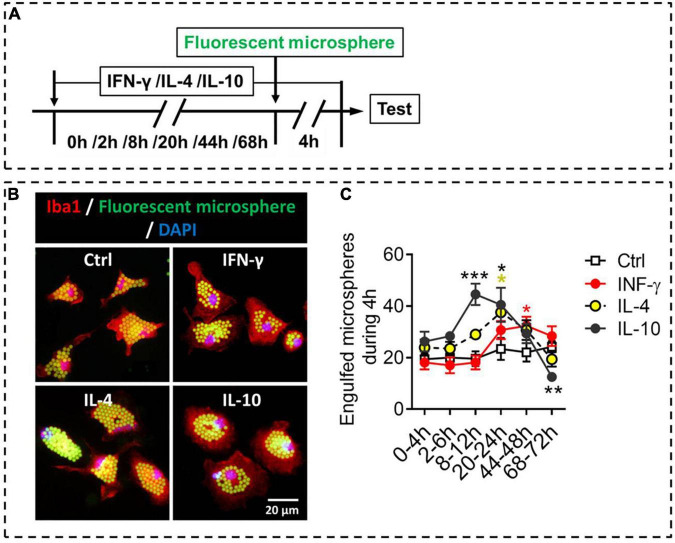
Phagocytic ability of microglial phenotypes induced by IFN-γ, IL-4, or IL-10. **(A)** Experimental scheme to monitor phagocytic ability during activation. Microglia underwent stimulation with IFN-γ, IL-4, or IL-10 for 0–68 h, then fluorescent microspheres were added and incubated with microglia for 4 h. **(B)** Representative micrographs showing immunocytochemical labeling for Iba1 (red) and engulfed fluorescent microspheres in microglia treated with IFN-γ, IL-4, or IL-10. **(C)** Quantification of the number of engulfed fluorescent microspheres in microglia during 4 h after treatment with IFN-γ, IL-4, or IL-10 for 2–72 h. Five, 40× micrographs were taken from each well, and all cells in the field were measured. Data are presented as mean ± SEM (*n* = 5–6 well per group), *P < 0.05, ***P < 0.005 (one-way ANOVA with Tukey’s multiple-comparisons test).

Like macrophages, microglia can phagocytose hazardous substances to maintain homeostasis; these substances include cell debris, beta-amyloid, and myelin. We compared the different phenotypes of stimulated microglia in terms of phagocytosis, an important function of microglia (Figure 6A). The results showed that IFN-γ promoted phagocytic ability of microglia at 44–48 h. IL-4 increased phagocytic ability of microglia at 20–24 h, after which it declined. Interestingly, IL-10 promoted phagocytic ability of microglia at 8–24 h, but inhibited phagocytic ability of microglia at 68–72 h (Figures 6B,C).

These findings suggest that IFN-γ, IL-4, and IL-10 differentially regulate phagocytic ability of primary microglia, which depends on exposure time in the stimulating molecule.

### Neurotoxicity of Primary Microglia Stimulated by IFN-γ, IL-4, or IL-10

**FIGURE 7:**
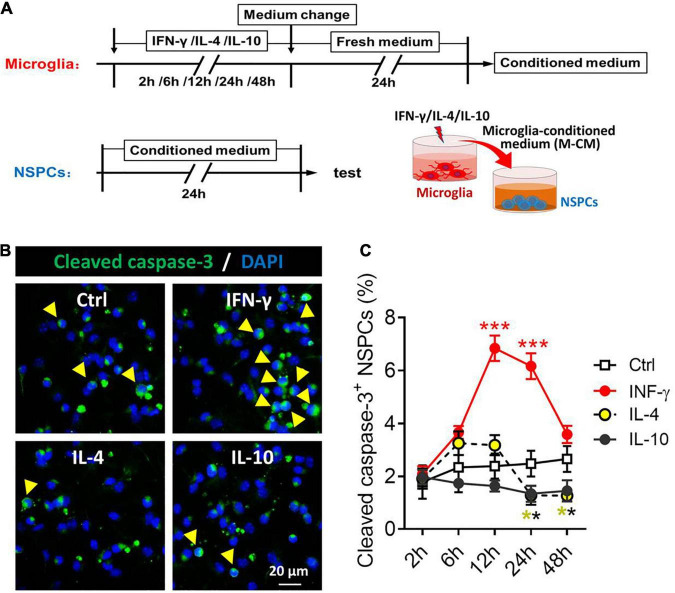
Neurotoxicity of microglial phenotypes induced by IFN-γ, IL-4, or IL-10. **(A)** Experimental scheme to monitor neurotoxicity during activation. Microglia underwent stimulation with IFN-γ, IL-4, or IL-10 for 2–48 h, then the medium was changed and the cells were cultured with fresh medium for 24 h. The microglia-conditioned medium (M-CM) was collected and configured as a proliferation medium for neural stem/precursor cells (NSPCs). The NSPCs were cultured with M-CM for 24 h and the apoptotic NSPCs were detected. **(B)** Representative micrographs showing immunocytochemical labeling for cleaved caspase-3 in NSPCs treated for 24 h with conditioned medium from IFN-γ-, IL- 4-, or IL-10-stimulated microglia. **(C)** Quantification of the percentages of cleaved caspase-3^+^-DAPI^+^ cells following treatment with conditioned medium from IFN-γ-, IL-4- or IL-10-stimulated microglia for 2–72 h. Five, 40× micrographs were taken from each well, and all cells in the field were measured. Data are presented as mean ± SEM (*n* = 4–6 well per group), **P < 0.01, ***P < 0.005 (one-way ANOVA with Tukey’s multiple-comparisons test).

Considering the neurotoxicity of different phenotypes of microglia, we compared the effects of microglia stimulated by IFN-γ, IL-4, or IL-10 and their secreted cytokines and soluble factors on the NSPCs (Figure 7A). The NSPCs were treated with conditioned medium from IFN-γ-, IL- 4-, or IL-10-treated microglia, and the neurotoxicity of microglia was assessed by quantifying the number of cleaved caspase-3^+^ NSPCs (Figure 7B). The results showed that the conditioned medium from IFN-γ-treated microglia increased the number of cleaved caspase-3^+^ NSPCs at 12–24 h. Conversely, the conditioned medium from IL-4- or IL-10-treated microglia decreased the number of cleaved caspase-3^+^ NSPCs at 24–48 h (Figure 7C).

These findings suggest that the neurotoxic or neuroprotective effect of microglia on NSPCs depended on a distinct phenotype. IFN-γ-stimulated microglia is neurotoxic to NSPCs, while the IL-4- or IL-10-stimulated microglia has neuroprotective effects on NSPCs.

### Plasticity of Microglial Phenotypes Based on Gene Profiling

**FIGURE 8:**
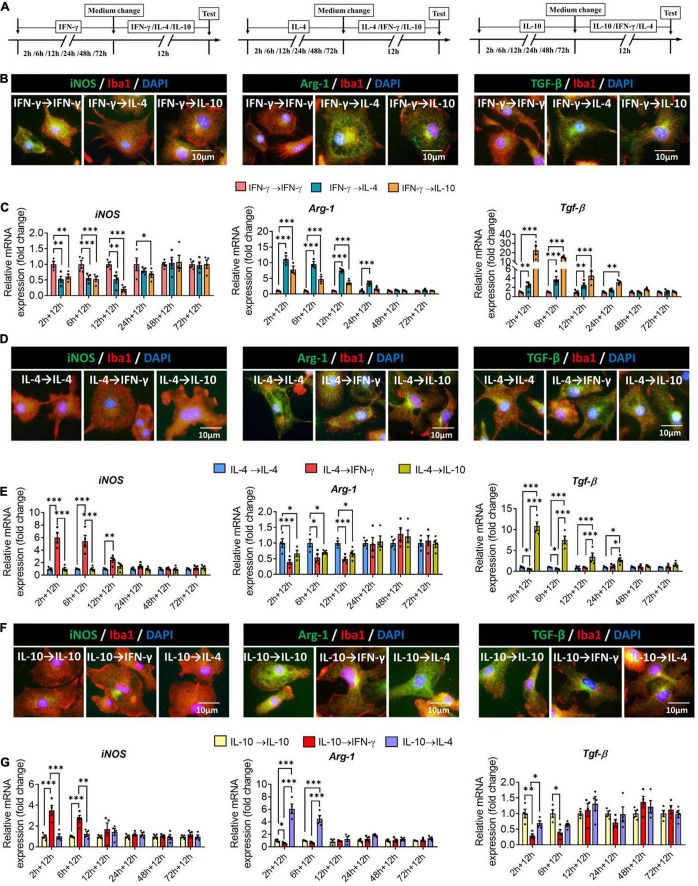
Plasticity of microglial phenotypes based on marker gene expression. **(A)** Experimental scheme. Microglia underwent primary stimulation with one cytokine for 2–72 h, then the medium was changed and the cells were stimulated with another cytokine for 12 h. Control cells were re-stimulated with the same cytokine as initially. **(B)** Fluorescence micrographs of primary microglial cultures after they were switched from the primary stimulation to secondary stimulation lasting 12 h. Cells were immunostained against Iba1 (red) and iNOS, Arg-1 or TGF-β (green). **(C)** Expression of iNOS, Arg-1 and TGF-β in microglia initially stimulated with IFN-γ, then stimulated with another cytokine. **(D)** Fluorescence micrographs of primary microglial cultures after they were switched from the primary IL-4 stimulation to secondary stimulation with IFN-γ or IL-10 lasting 12 h. Cells were immunostained against Iba1 (red) and iNOS, Arg-1 or TGF-β (green). **(E)** Expression of iNOS, Arg-1 and TGF-β in microglia initially stimulated with IL-4, then stimulated with another cytokine. **(F)** Immunocytochemical double-labeling for Iba1 (red) and iNOS, Arg-1 or TGF-β (green) in IL-10-stimulated microglia exposed to IFN-γ or IL-4 for 12 h. Fluorescence micrographs of primary microglial cultures after they were switched from the primary IL-10 stimulation to secondary stimulation with IFN-γ or IL-4 lasting 12 h. Cells were immunostained against Iba1 (red) and iNOS, Arg-1 or TGF-β (green). **(G)** Expression of iNOS, Arg-1, and TGF-β in microglia initially stimulated with IL-10, then stimulated with another cytokine. Data are presented as mean ± SEM (*n* = 4 well per group, three replicates per sample), *P < 0.05, ^**^P < 0.01, ^***^P < 0.005 (one-way ANOVA with Tukey’s multiple-comparisons test).

To examine whether primary microglia already induced to adopt one phenotype could be switched to another phenotype, we exposed cells to one of the three cytokines for 2–72 h, after which a different cytokine was applied for another 12 h (Figure 8A). When microglia were exposed to IFN-γ for shorter than 24 h, subsequent IL-4 treatment downregulated immune defense-related iNOS and upregulated immunomodulation-related Arg-1, whereas IL-10 treatment downregulated iNOS and upregulated immune deactivation-related TGF*-*β (Figures 8B,C).

When microglia were exposed to IL-4 for shorter than 12 h, subsequent IFN-γ treatment upregulated immune defense-related iNOS and downregulated immunomodulation-related Arg-1.

When microglia were exposed to IL-4 for shorter than 24 h, subsequent IL-10 treatment upregulated immune deactivation-related TGF-β (Figures 8D,E).

When microglia were exposed to IL-10 for shorter than 6 h, subsequent IFN-γ treatment upregulated iNOS and downregulated TGF-β, while IL-4 treatment upregulated Arg-1 (Figures 8F,G).

These results suggest that even though microglia are induced into different phenotypes by specific cytokines, its gene profiling is highly malleable. The immunodefensive phenotype of microglia induced by IFN-γ is switched more easily, while the immunosuppressive phenotype of microglia induced by IL-10 is inflexible, and it’s easier for the immunoregulatory phenotype of microglia induced by IL-4 to turn immunosuppressive phenotype than immunodefensive phenotype.

### Plasticity of Microglial Phenotypes Based on Morphology

**FIGURE 9:**
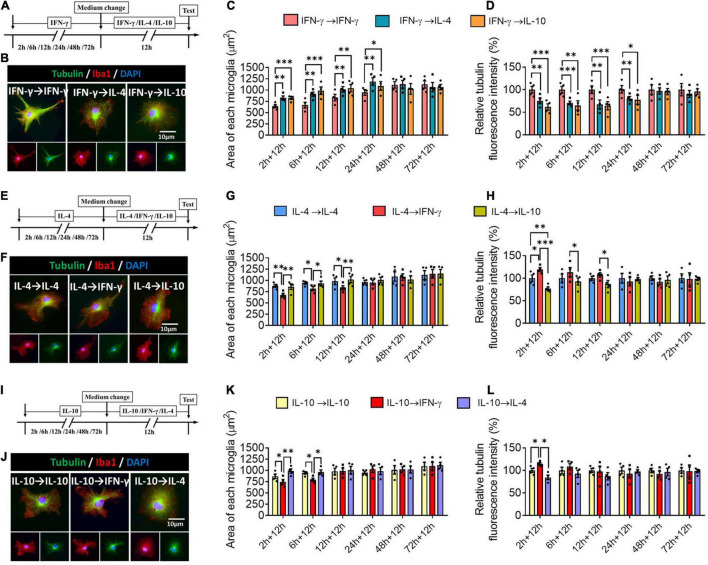
Plasticity of microglial phenotypes based on morphology. **(A–D)** Plasticity of morphology of IFN-γ-treated microglia in different response state following IL-4 or IL-10 exposure for 12 h. **(A)** Experimental scheme of IFN-γ-stimulated microglia at 2, 4, 6, 12, 24, 48, and 72 h following IL-4 or IL-10 exposure. Same steps as Figure 8A. **(B)** Fluorescence micrographs of microglial cytoskeleton changes after cells were switched from the primary IFN-γ stimulation to secondary stimulation with IL-4 or IL-10 lasting 12 h. Cells were immunostained against tubulin (green) and Iba1 (red). **(C)** Quantification of the area in microglia initially stimulated with IFN-γ, then stimulated with another cytokine. **(D)** The changes in relative tubulin fluoresce intensity in microglia initially stimulated with IFN-γ, then stimulated with another cytokine. **(E–H)** Plasticity of morphology of IL-4-treated microglia in different response state following IFN-γ or IL-10 exposure for 12 h. **(I–L)** Plasticity of morphology of IL-10-treated microglia in different response state following IFN-γ or IL-4 exposure for 12 h. Five 40× photographs were taken from each well, and all the cells in the field were measured. Data are presented as the mean ± SEM. *n* = 4 well per group, *P < 0.05, ^**^P < 0.01, ^***^P < 0.005 (one-way ANOVA with Tukey’s multiple-comparisons test).

Next we assessed the plasticity of the different microglial phenotypes in terms of morphology. The microglial morphology and cytoskeleton induced by IFN-γ were significantly altered during 24-h treatment with IL-4 or IL-10, leading to an increase in microglial area but a decrease in the density of tubulin. When microglia were exposed to IFN-γ for longer than 24 h, subsequent treatment with either IL-4 or IL-10 did not significantly alter their morphology (Figures 9A–D). When microglia were exposed to IL-4 for shorter than 12 h, IFN-γ, but not IL-10, significantly reduced microglial area and increased tubulin density. When microglia were exposed to IL-4 for longer than 12 h, their morphology was unaltered by either of the other cytokines, even after 72 h (Figures 9E–H). When microglia were exposed to IL-10 for shorter than 6 h, IFN-γ but not IL-4 significantly reduced microglial area and increased tubulin density (Figures 9I–L).

These morphological findings support that the immunodefensive and immunoregulatory microglia has obvious morphological plasticity.

### Plasticity of Microglial Phenotypes Based on Proliferative Ability

**FIGURE 10:**
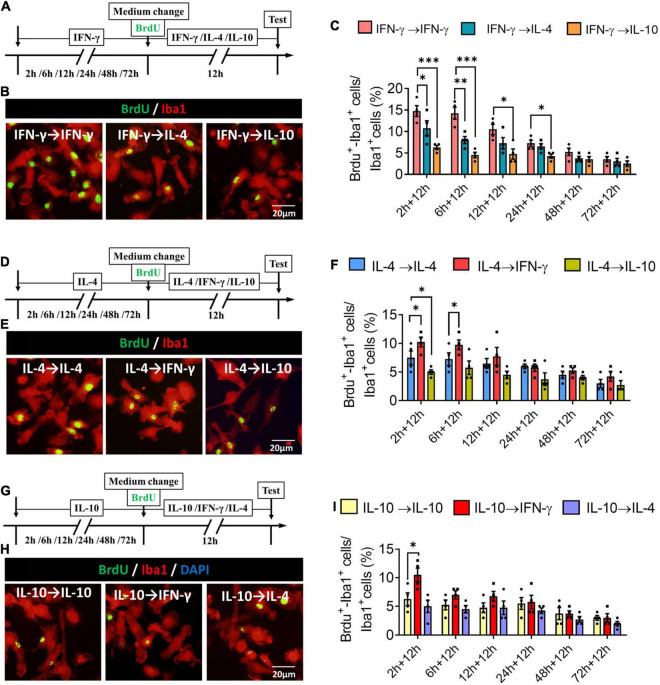
Plasticity of proliferation ability of IFN-γ, IL- 4-, or IL-10-treated microglia in different response state. **(A–C)** Plasticity of proliferation ability of IFN-γ-treated microglia in different response state. **(A)** Experimental protocol for monitoring proliferation capacity ability after microglia initially stimulated with IFN-γ for 2–72 h, then stimulated with another cytokine for 12 h. **(B)** Fluorescence micrographs of primary microglial cultures after they were switched from the primary IFN-γ stimulation to secondary stimulation with IL-4 or IL-10 lasting 12 h. Cells were immunostained against Iba1 (red) and BrdU (green). **(C)** Percentage of proliferating microglia initially stimulated with IFN-γ, then stimulated with another cytokine. **(D–F)** Plasticity of proliferation ability of IL-4-treated microglia in different response state following IFN-γ or IL-10 exposure for 12 h. **(G–I)** Plasticity of proliferation ability of IL-10-treated microglia in different response state following IFN-γ or IL-4 exposure for 12 h. Five 40× photographs were taken from each well, and all the cells in the field were measured. Data are presented as the mean ± SEM. *n* = 4–5 well per group, *P < 0.05, ^**^P < 0.01 (one-way ANOVA with Tukey’s multiple-comparisons test).

When microglia were exposed to IFN-γ for shorter than 6 h, subsequent IL-4 treatment significantly decreased the number of BrdU^+^-Iba1^+^ cells. When microglia were exposed to IFN-γ for shorter than 24 h, subsequent IL-10 treatment also decreased the number of BrdU^+^-Iba1^+^ cells (Figures 10A–C). When microglia were exposed to IL-4 for shorter than 6 h, subsequent IFN-γ treatment significantly increased the number of BrdU^+^-Iba1^+^ cells. When microglia were exposed to IL-4 for shorter than 2 h, subsequent IL-10 treatment decreased the number of BrdU^+^-Iba1^+^ cells (Figures 10D–F). Conversely, when microglia were exposed to IL-10 for shorter than 2 h, subsequent IFN-γ treatment increased the number of BrdU^+^-Iba1^+^ cells (Figures 10G–I).

These findings for proliferative ability support that the immunodefensive and immunoregulatory microglia has obvious plasticity in self-proliferation.

### Plasticity of Microglial Phenotypes Based on Phagocytic Ability

**FIGURE 11:**
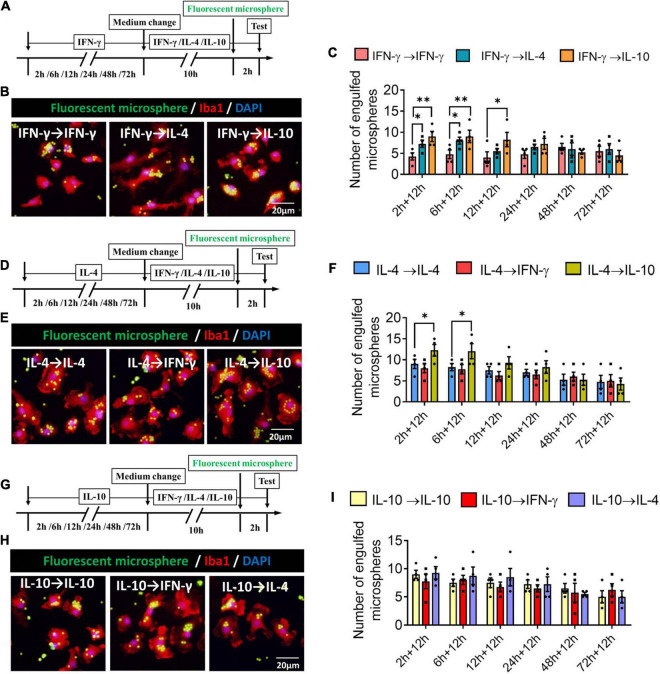
Plasticity of phagocytic ability of IFN-γ, IL- 4-, or IL-10-treated microglia in different response state. **(A–C)** Plasticity of phagocytic ability of IFN-γ-treated microglia in different response state. **(A)** Experimental design. IFN-γ-stimulated microglia at different reaction stages following IL-4 or IL-10 exposure for 12 h. Phagocytic activity was measured in the last 2 h. **(B)** Immunofluorescence imaging was used to examine the phagocytic activity of microglia. **(C)** Quantification of number of engulfed microsphere by each microglia. **(D–F)** Plasticity of phagocytic ability of IL-4-treated microglia in different response state following IFN-γ or IL-10 exposure for 12 h. **(G–I)** Plasticity of phagocytic ability of IL-10-treated microglia in different response state following IFN-γ or IL-4 exposure for 12 h. Five 40× photographs were taken from each well, and all the cells in the field were measured. Data are presented as the mean ± SEM. *n* = 4–5 well per group, *P < 0.05, ^**^P < 0.01 (one-way ANOVA with Tukey’s multiple-comparisons test).

We found that when microglia were exposed to IFN-γ for shorter than 6 h, subsequent IL-4 treatment significantly increased the number of engulfed microspheres. When microglia were exposed to IFN-γ for shorter than 12 h, subsequent IL-10 treatment increased the number of engulfed microspheres (Figures 11A–C). When microglia were exposed to IL-4 for shorter than 6 h, subsequent IL-10 treatment increased the number of engulfed microspheres. IFN-γ treatment did not alter the phagocytic ability of IL-4-stimulated microglia at any time (Figures 11D–F). Treatment with IFN-γ or IL-4 did not alter the phagocytic ability of IL-4-stimulated microglia at any time (Figures 11G–I).

These findings for phagocytic ability support that the immunodefensive and immunoregulatory microglia has obvious plasticity in phagocytosis.

### Plasticity of Microglial Phenotypes Based on Neurotoxicity or Neurotrophy

**FIGURE 12:**
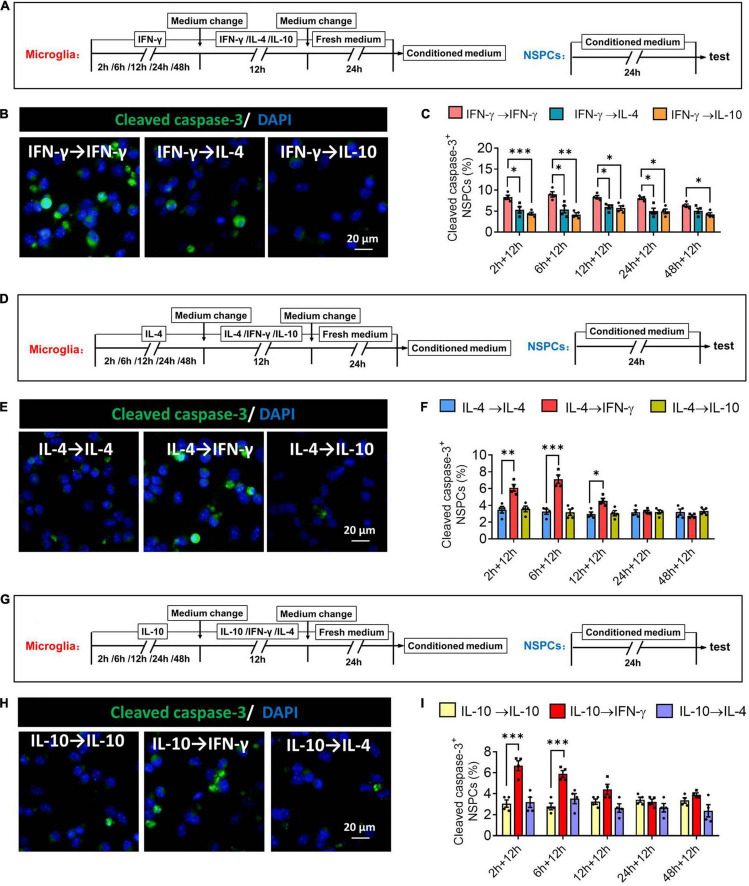
Changes in neurotoxicity of IFN-γ, IL- 4-, or IL-10-treated microglia in different response state. **(A–C)** Changes in neurotoxicity of IFN-γ-treated microglia on NSPCs in different response state following IL-4 or IL-10 exposure for 12 h. **(A)** Experimental scheme to monitor neurotoxicity during activation. Microglia underwent stimulation with IFN-γ, IL-4, or IL-10 for 2–48 h, then the medium was changed and the cells were stimulated with another cytokine for 12 h. After then the cells were cultured with fresh medium for 24 h. The microglia-conditioned medium (M-CM) was collected and configured as a proliferation medium for neural stem/precursor cells (NSPCs). The NSPCs were cultured with M-CM for 24 h and the apoptotic NSPCs were detected. **(B)** Representative micrographs showing immunocytochemical labeling for cleaved caspase-3 in NSPCs treated for 24 h with conditioned medium from the reprogrammed microglia. **(C)** Quantification of the percentages of cleaved caspase-3^+^-DAPI^+^ cells following treatment with conditioned medium from the reprogrammed microglia. **(D–F)** Changes in neurotoxicity of IL-4-treated microglia on NSPCs in different response state following IFN-γ or IL-10 exposure for 12 h. **(G–I)** Changes in neurotoxicity of IL-10-treated microglia on NSPCs in different response state following IFN-γ or IL-10 exposure for 12 h. Five 40× photographs were taken from each well, and all the cells in the field were measured. Data are presented as the mean ± SEM. *n* = 4–5 well per group, *P < 0.05, ^**^P < 0.01, ^***^P < 0.005 (one-way ANOVA with Tukey’s multiple-comparisons test).

When microglia were exposed to IFN-γ for shorter than 24 h, subsequent IL-4 treatment significantly decreased the number of cleaved capase-3^+^ NSPCs. When microglia were exposed to IFN-γ for shorter than 48 h, subsequent IL-10 treatment also decreased the number of cleaved capase-3^+^ NSPCs (Figures 12A–C). When microglia were exposed to IL-4 for shorter than 12 h, subsequent IFN-γ treatment significantly increased the number of cleaved capase-3^+^ NSPCs (Figures 12D–F). Conversely, when microglia were exposed to IL-10 for shorter than 6 h, subsequent IFN-γ treatment increased the number of cleaved capase-3^+^ NSPCs (Figures 12G–I).

These findings suggest that the neurotoxicity of immunodefensive microglia induced by IFN-γ could be weakened through IL-4 or IL-10 reprogramming.

## Discussion

**FIGURE 13:**
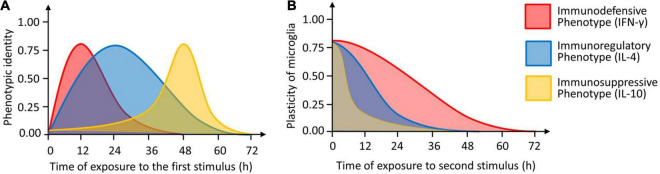
The identity and plasticity of microglial phenotype. **(A)** Timing of induction of three microglial phenotypes (immunodefensive phenotype induced by IFN-γ, immunoregulatory phenotype induced by IL-4 and immunosuppressive phenotype induced by IL-10). The “1.0” means that the cells show the maximal identify. **(B)** Plasticity of microglial phenotypes as a function of the length of second stimulation. The “1.0” means that the cells show the maximal plasticity.

Microglia are a single cell type with exceptional plasticity, but relatively little is known about how specific stimuli induce specific phenotypes or about the precise morphological or functional characteristics of each phenotype. Here we used expression profiling and analysis of morphology, proliferation, phagocytosis and neurotoxicity to define three potential microglial phenotypes induced by three cytokines. Our experiments suggest that IFN-γ induces an immunodefensive phenotype; IL-4, an immunoregulatory phenotype; and IL-10, an immunosuppressive phenotype (Figure 13).

IFN-γ is a Th1 cytokine that plays an important role in early immunological responses to viral infection and tumors, and it can induce microglial priming (Spencer et al., 2016; Prajeeth et al., 2018; Ta et al., 2019). Elevated IFN-γ concentrations have been found in various neurodegenerative diseases and psychiatric disorders, and we have shown that IFN-γ can activate microglia by stimulating the JAK/STAT pathway to impair adult hippocampal neurogenesis and cause depression-like behaviors and cognitive defects (Mount et al., 2007; Moritz et al., 2017; Zhang et al., 2020a). Here we found that IFN-γ up- and downregulated 332 genes, most of which are involved in immune defense and inflammatory responses. The most strongly upregulated DEGs encode iNOS and TNF-α. The enzyme iNOS catalyzes the synthesis of NO and citrulline from L-arginine. Both iNOS and TNF-α regulate nearly all phases of immune and inflammatory responses. We found that IFN-γ upregulated both genes from 2 to 24 h, after which expression returned to baseline. These data suggest that IFN-γ induces an acute pro-inflammatory microglial phenotype that self-resolves to control inflammation. The upregulation of these genes correlated with enlargement of the soma and extension of multiple filopodia, which developed into lamellipodia. The extension of multiple filopodia has been associated with immune alert. The upregulation of iNOS and TNF-α also correlated with increased proliferative ability and neurotoxicity. Taken together, these results suggest that IFN-γ induces an immunodefensive phenotype in microglia. Our results may help explain how IFN-γ induces so-called “microglial priming,” which exaggerated immune responses upon stimulation with microbial or endogenous ligands, such as bacterial lipopolysaccharide (LPS) or amyloid-β peptide.

IL-4 is a Th2 cytokine involved in the regulation of inflammatory responses and physiological processes of the central nervous system (Gadani et al., 2012). Our previous studies have shown that IL-4 can reprogram microglia toward a neuroprotective phenotype for maintaining brain homeostasis and promoting neurogenesis and tissue repair (Zhang et al., 2021). Here we showed that IL-4 up- or downregulated 432 genes, most of which are involved in immunomodulation. The most strongly upregulated genes encode Arg-1 and Ym-1. Arg-1 is a manganese metalloenzyme that hydrolyzes L-arginine to L-ornithine, opposing the activity of iNOS. In our experiments, IL-4 persistently upregulated Arg-1 during 48 h, which correlated with the extension of several long lamellipodia, which contribute to immunoregulation. It also correlated with an increase in phagocytosis, which plays an important role in maintenance of brain homeostasis (Fu et al., 2014; Yi et al., 2020). Disorders of microglial phagocytosis are implicated in many neurological diseases such as Alzheimer’s disease, multiple sclerosis, Parkinson’s disease, traumatic brain injury ischemic and other brain diseases (Fu et al., 2014). Upregulation of IL-4 expression in the hippocampus of APP/PS1 mice promoted β-amyloid (Aβ) clearance by microglia and ameliorated cognitive deficits (Kiyota et al., 2010). Previous work has shown that IL-4 activates STAT6 in microglia, in turn activating PPARγ and shifting the cells toward an immunomodulation-related phenotype (Li et al., 2014). Future work should investigate the role of STAT6 and other signaling molecule in this phenotype.

IL-10 is an immunosuppressive cytokine with anti- inflammatory properties that can prevent inflammation-mediated neuronal degeneration (Porro et al., 2020). In previous studies, we also found that IL-10 inhibits microglia-mediated CNS inflammation in hippocampus, suggesting that it may have antidepressant effects (Zhang et al., 2016, 2017). Here we showed that IL-10 up- or downregulated 89 genes, most of which are involved in immunosuppression and production of TNF-related apoptosis-inducing ligand. The most strongly upregulated genes encode TGF-β and IL-10. TGF-β, which is secreted mainly by microglia, regulates proliferation and differentiation of various nerve cells, and its secretion is enhanced by apoptotic cells (Butovsky et al., 2014). In our experiments, IL-10 upregulated TGF-β in microglia only after 24 h, when microglia retracted their processes and became amoeboid. Such processes have been associated with immunosuppressive and tissue repair. In addition, IL-10 inhibited microglial proliferation. Previous studies have shown that IL-10 activates STAT3 and SOCS3, shifting microglia toward an immunosuppressive state in which M2c markers are upregulated (Guillot-Sestier et al., 2015; Weston et al., 2021). Future work should investigate the role of STAT3, SOCS3, and other signaling molecules in this phenotype.

Studies have suggested that microglial phenotype is quite malleable: for example, pro-inflammatory microglia can be induced to switch to an anti-inflammatory phenotype, which helps contain inflammation (Zhang et al., 2018). CNS inflammation can be completely or partially prevented by resting microglia performing anti-inflammatory therapy in advance of inflammatory stimulation, which helps prevent damage caused by CNS inflammation (Han et al., 2015; Zhang et al., 2017). What is more interesting is that microglia were stimulated with both pro-inflammatory and anti-inflammatory molecules, inducing hybrid phenotype with a variety of phenotypic characteristics (Gomez-Nicola and Perry, 2015; Yi et al., 2020). Consistent with this, we showed here that microglia induced to adopt each of the three phenotypes could be switched to adopt either of the other two phenotypes, simply by changing the stimulating cytokine. Microglia phenotypic characteristics (such as gene expression, morphology, and proliferative capacity) and functions (such as phagocytosis, immune response, and neurotoxicity) also show significant plasticity. Notably, plasticity decreased with time. IL-4-stimulated microglia remained capable of switching phenotype within 12 h, while IFN-γ-stimulated microglia remained malleable until 24 h. IL-10-stimulated microglia, in contrast, showed much lower plasticity than the other phenotypes, regardless of the length of the first stimulation. A large variety of receptors are present in microglia, whose activation by immune signals, neurotransmitters, neuropeptides, hormones, lipid messengers, metabolites, and even alimentary components drives phenotypic transition of microglia (Gomez-Nicola and Perry, 2015). Studies showed that interferon regulatory factor 8 (IRF8) is a key constitutive determinant of the morphological and molecular properties and motility of microglia in the central nervous system (Horiuchi et al., 2012; Masuda et al., 2014). While the nuclear receptor PPAR-γ, a critical transcription factor for transforming microglia into a neuroprotective phenotype, alleviating a variety of brain diseases, such as depression, stroke, Alzheimer’s disease, and so on (Pan et al., 2015; Zhao et al., 2016; Xie et al., 2020; Zhang et al., 2020b). These findings support emerging strategies able to redirect microglia from detrimental to beneficial functions, and open novel approaches to target microglia therapeutically.

Due to the complexity of the brain’s internal environment, which includes several interacting cell populations and soluble factors, microglia behave very differently *in vitro* versus *in vivo* (Zhang et al., 2020c). In primary culture, most microglial cells exhibit ameboid morphology without branches, while some have several simple branches (Giulian and Baker, 1986; Figarella et al., 2018). In contrast, microglia in the brain have a more complex branching structure, characterized by multiple branches protruding from small somata (Sankowski et al., 2019; Zhang et al., 2020c). Our previous studies have shown that astrocytes play an important role in regulating microglial ramification and function (Zhang et al., 2020c). Studies in mouse have applied transcriptome-wide profiling of microglia to reveal key features of microglial ontogeny, functional profile, and phenotypic diversity. While similar, human microglia exhibit clear differences to their mouse counterparts (Patir et al., 2019). This study was limited in exploring the plasticity of morphology, molecular properties and function in mouse primary microglia *in vitro*. Subsequent research need to develop a better understanding of the mouse and human microglial profile *in vivo*.

## Conclusion

We have revealed that microglia are highly plastic in morphology, phenotypic profile, proliferative capacity, and phagocytic ability after stimulation with different cytokines. This plasticity declines or even disappears with longer stimulation, implying that chronic activation of microglia may lead to irreversible damage in many neurological diseases. Our results may help explain how microglia are able to adopt such diverse functions in health and disease.

## Data Availability Statement

The datasets presented in this study can be found in online repositories. The names of the repository/repositories and accession number(s) can be found below: http://www.ncbi.nlm.nih.gov/bioproject/787892.

## Ethics Statement

The animal study was reviewed and approved by Institutional Animal Care and Use Committee of the Guizhou University of Traditional Chinese Medicine.

## Author Contributions

JZ and XJ conceived and designed the study and wrote the manuscript. LM and HH performed the primary microglial culture and testing for phenotypic markers. QL, SY, LL, and DS performed the neural stem/precursor cell analysis, phagocytosis and neurotoxicity tests, immunofluorescence studies, and cytokine assays. FY and YZ performed statistical analysis. All authors approved the final version of the manuscript.

## Conflict of Interest

The authors declare that the research was conducted in the absence of any commercial or financial relationships that could be construed as a potential conflict of interest.

## Publisher’s Note

All claims expressed in this article are solely those of the authors and do not necessarily represent those of their affiliated organizations, or those of the publisher, the editors and the reviewers. Any product that may be evaluated in this article, or claim that may be made by its manufacturer, is not guaranteed or endorsed by the publisher.

## Abbreviations

Arg-1: arginase-1
BrdU: bromodeoxyuridine
DAPI: 4′,6-diamidino-2-phenylindole
DEGs: differentially expressed genes
GO: Gene Ontology
IFN-γ: interferon-gamma
IL-4: interleukin-4
IL-10: interleukin-10
iNOS: inducible nitric oxide synthase
IL-1β: interleukin-1 beta
IL-6: interleukin-6
IRFs: regulating interferon regulatory factors
Iba1: ionized calcium binding adapter molecule 1
KEGG: Kyoto Encyclopedia of Genes and Genomes
M-CM: microglia-conditioned medium
NSPCs: neural stem/progenitor cells
NO: nitric oxide
PBS: phosphate-buffered saline
PFA: paraformaldehyde
SVZ: subependymal ventricular zone
TNF-α: tumor necrosis factor alpha
TGF-β: transforming growth factor-beta.
